# Correlation Between Subclinical Hypothyroidism and Dyslipidemia

**Published:** 2017-04-01

**Authors:** Alireza Rastgooye Haghi, Mahdis Solhjoo, Mohammad Hossein Tavakoli

**Affiliations:** 1 *Dept. of Medical Science of Hamadan University of Medical sciences, Hamedan, Iran*

**Keywords:** Subclinical Hypothyroidism, Dyslipidemia, Thyroid

## Abstract

**Background & Objective:**

Thyroid hormones have an important role in the regulation of lipid metabolism. Subclinical hypothyroidism (SCH), defined as a mild increase in thyroid-stimulating hormone (TSH) and normal level of thyroxine (T4), could be associated with altered lipid profile. The current study aimed at assessing the association between SCH and changes in lipid profile.

**Methods:**

Data of 53 patients with SCH and 53 euthyroid cases were collected from Besat Hospital in Hamadan, Iran, in 2013. The age range of the cases was 18 to 60 years, and the groups were matched in terms of gender, age, and body mass index (BMI). SCH was defined as a TSH value of 4.2 to 10 mU/L, and normal T4 as 0.8 to 2.8 ng/dL. Control cases had a normal TSH ranging from 0.5 to 4.2 mU/L. The total serum cholesterol (TCHOL), high-density lipoprotein (HDL) cholesterol, low-density lipoprotein (LDL) cholesterol, and triglyceride (TG) levels in both groups were examined and the results were recorded.

**Results:**

Participants with SCH had significantly higher LDL and lower HDL levels than the control group regardless of age group and gender (P-value <0.001), but there was no difference in TG and TCHOL levels (P-value <0.05). The prevalence of dyslipidemia and SCH was only significant in females (P-value =0.009). Totally, there was significant correlation between the prevalence of dyslipidemia and SCH regardless of gender (P-value =0.04).

**Conclusion:**

SCH is associated with dyslipidemia, and biochemical screening for thyroid dysfunction is recommended in all patients with dyslipidemia.

## Introduction

Thyroid gland is an endocrine gland at the anterior of trachea between cricoid cartilage and suprasternal notch (1). This gland produces 2 important hormones namely thyroxine (T4) and triiodothyronine (T3) that exert their effects through the nuclear receptors. They play a critical role in cell differentiation during growth and development. They also maintain metabolic homeostasis in body (1). Thyroid disorders are often due to autoimmune processes that result in the increase (hyperthyroidism) or decrease of thyroid hormones (hypothyroidism) (1). The average annual incidence of autoimmune hypothyroidism is 4 cases per 1000 females and 1 case per 1000 males (1). Since autoimmune reactions reduce thyroid functions gradually, the first stages of hypothyroidism are compensated by an increase in thyroid-stimulating hormone (TSH), which maintains T4 in the normal range (2). This level is mostly observed in the initial stages of Hashimoto's disease called subclinical hypothyroidism (SCH). Patients at this stage have no symptoms or the symptoms are very mild. When the SCH is associated with antibodies against thyroid peroxidase (TPO), annual risk of developing overt hypothyroidism is about 4% (1,2).

Hyperlipidemia is one of the components of metabolic syndrome. In some studies, metabolic syndrome and its components (dyslipidemia) are responsible for 25%of the new onset cardiovascular disease (CVD) (3). Thyroid hormones have an important regulatory effect on glucose and lipid metabolism, and blood pressure control. Increasing in total cholesterol and low-density lipoprotein (LDL) in hypothyroidism might be due to several changes in the synthesis, metabolism, and fat mobilization (3). Thyroid hormones increase 3-hydroxy-3-methyl-glutaryl-coenzyme A (HMG-COA) reductase activity in the liver and, thus, reduce cholesterol. In addition, thyroid hormones increase LDL receptors on fibroblasts, liver, and other tissue, and they increase absorption of cholesterol from the intestine. These hormones also alter levels of high-density lipoprotein (HDL) cholesterol and hepatic lipase activity, and affect the excretion of cholesterol from the intestine by bile acids (3). While the relationship between subclinical hypothyroidism and an increased risk of cardiovascular disease caused by atherosclerosis is shown in some studies, it is not confirmed in the other studies (3, 4). Although the effects of subclinical hypothyroidism on serum lipids are unclear, it is likely that the changes that occur in clinical hypothyroidism, exist in subclinical cases as well, but might be much less severe. Due to the role of dyslipidemia as an important risk factor of atherosclerosis, which may appear in subclinical hypothyroidism, the current study aimed at assessing the prevalence of dyslipidemia in patients with subclinical hypothyroidism, in comparison with healthy people in Hamadan, Iran.

## Material and Methods

The current cross sectional study was performed at Endocrinology Clinic of Besat Hospital in Hamadan, Iran, in 2013; 53 patients with subclinical hypothyroidism (10 mU/L > TSH >4.2 mU/L) and normal T4 (0.8 to 2.8 ng/dL), measured twice with one month interval without clinical symptoms, were selected. Also, 53 euthyroid participants with normal levels of TSH, ranged from0.5 to 4.2 mU/L were enrolled into the study as the control group. The age range of the cases was 18 to 60 years. The control group was matched with the patient group in terms of gender, age, and body mass index (BMI). For any patient with elevated TSH, the following causes were considered: Use of dopaminergic drugs, amiodarone, lithium, drugs containing iodine, circadian TSH secretion (TSH level decreases in the morning and increases in the evening), euthyroid outliers (25% of healthy people with TSH higher than normal range), and sick euthyroid syndrome. Also, patients with primary and secondary risk factors for hyperlipidemia such as history of familial hyperlipidemia, obesity, diabetes, kidney disease, and steroid users were excluded from the study. Therefore, after taking a full medical history of acute recent illnesses and previous backgrounds, patients were enrolled into the study. All thyroid panel tests were performed based on enzyme-linked fluorescent assay (ELFA) by Vitek immunodiagnostic assay system (VIDAS). For ethical purpose, each patient signed the letter of informed consent. The following procedures were in accordance with the ethical standards of the institutional or regional responsible committee on human experimentation and those of the Helsinki Declaration of 1975, as revised in 1983.

A total of 10 mL fasting venous blood samples were taken from each case in early morning to measure lipid profiles, including triglyceride (TG), HDL, LDL,, and total cholesterol (TCHOL). Dyslipidemia in Besat Hospital laboratory (according to the Harrison principles of internal medicine (1) is defined as TG >200 mg/dL, TCHOL >200 mg/dL, LDL >100 mg/dL, HDL <40 mg/dL in males and HDL <50 mg/dL in females. All the lipid profile tests were done in Besat Hospital laboratory by enzymatic methods using the autoanalyzer BT. The data and the tests results of the 2 groups were collected and recorded in the checklist blindly. For data analysis, statistical methods such as Chi-student *t* test and one-sample *t* test were used. All comparisons were based on the presence of a significant correlation with P-values <0.05 and the confidence interval (CI) of 95%. 

## Results

In the current study, the age range of all the 53 cases with SCH, and the 53 cases as the control group was from 18 to 60 years. Among them, females were 57% and males were 49% in both groups. Table 1 shows the distribution of demographic characteristics among the study cases.

**Table 1:** Demographical Characteristics of the Study Cases

Parameters	Case Group Mean ±SD	Control Group Mean ±SD	P-value
Gender	25 M 28 F	24 M 29 F	P >0.05
Age (years)	45.4 11.2	47.2 11.75	P >0.05
Weight (kg)	70.15 10.39	71.989.91	P >0.05
Height (m)	1.71 0.91	1.72 0.74	P >0.05
BMI (kg/m^2^)	23.77 5.47	24.4 3.56	P >0.05

BMI, body mass index

In females, the TCHOL levels of the patients group were higher than those of the control group in all age groups, but it was significant only in the age group of 40 to 50 years (P-value =0.03). The results were the same in males (P-value =0.01).

There was significant correlation between SCH and decreased level of HDL in females regardless of age groups (P-value =0.001). Also, the results of the study showed a significant correlation between increased level of LDL and SCH in all age groups of females, except the group 30 to 40 years. In males, there was no significant correlation between SCH and decreased level of HDL, or increased level of LDL in all age groups. No significant correlation between increased level of TG and SCH was observed in the 2 genders. In overall, there was significant correlation between SCH and decreased HDL level (P-value = 0.04) and also increased LDL level (P-value =0.001).

Regardless of age groups and gender, there were no significant correlations between SCH and increased levels of TG and TCHOL (P-value <0.05). The prevalence of dyslipidemia and SCH was significant in females (P-value =0.009), but not in males (P-value =0.02). Totally, there was a significant correlation between the prevalence of dyslipidemia and SCH regardless of gender (P-value =0.04). Table 2 shows the correlation between SCH and lipid profiles based in age groups and gender in summary.

**Table 2:** The Correlation between SCH and Lipid Profiles Based on Age Groups and Gender

TCHOL		HDL		LDL		TG	
**Case**	Control	Case	Control	Case	Control	Case	Control
Age Groups (years)	**18-30**	F 152.67 47.59	F 131.67 56.22	F 71.83 26.17	F 90 25.6	F 128.17 47.94	F 70 22.15	F 129.83 46.05	F 121.83 37.81
		M 145.5 60.59	M 146.25 67.44	M 65 30.88	M 53 7.26	M 96 20.29	M 97.50 12.39	M 182 27.36	M 142.75 44.51
	**30-40**	F 161.57 29.79	F 150.56 24.51	F 62.71 25.09	F 73.75 27.46	F 95.86 38.63	F 80.81 20.14	F 162.86 48.72	F 138.13 50.48
		M 104.63 26.47	M 131.71 53.54	M 79.75 60.51	M 104 26.38	M 144.31 71.3	M 84.86 28.21	M 133.5 35.44	M 108.71 20.01
	**40-50**	F 179.04 65.4	F 131.46 31.4	F 40 9.15	F 78.46 27.92	F 104.25 26.06	F 72 23.59	F 164.25 79.21	F 135.08 47.21
		M 214.63 58.72	M 147.13 33.9	M 92.25 48.94	M 80 29.46	M 85 25.09	M 89.25 55.84	M 114.75 37.39	M 131.13 47.55
	**50-60**	F 157.33 49.74	F 203.23 27.62	F 41 7.81	F 42.6 10.07	F 180 22	F 73.33 8.02	F 100 39.85	F 175.33 50.93
		M 127.24 80.67	M 124.6 39.35	M 78.4 30.08	M 81.4 16.38	M 117.6 42.05	M 107.2 55.54	M 165.5 23.16	M 133.8 28.62
	**18-60**	F 166.7 75.25	F 144.17 40.52	F 52.61 22.35	F 75.84 31.87	F 115.39 41.52	F 74.16 20.76	F 149.64 63.99	F 137.34 46.50
		M 150.84 81.05	M 137.79 45.29	M 81.12 46.08	M 82.79 28.17	M 112.26 51.51	M 93.08 42.26	M 141.66 40.13	M 127.08 36.79
P Value		Non-significant		significant		significant		Non- significant	

F: Female; M: Male

* The significant relations have bolded frame

SCH, subclinical hypothyroidism; TCHOL, total serum cholesterol; HDL, high-density lipoprotein; LDL, low-density lipoprotein; TG, triglyceride

## Discussion

Hypothyroidism is a common endocrine disorder worldwide. Thyroid hormones have an important role in the regulation of lipid synthesis, absorption, and metabolism (5). Hypothyroidism is associated with significant increase in circulating concentrations of LDL, which can lead to coronary artery disease (5).

In some studies, even within the normal range of TSH levels, a linear increase in TCHOL, LDL, and TG, and a linear decrease in HDL levels were observed with increasing TSH (6). Subclinical hypothyroidism is the clinical status of elevated serum TSH levels with normal levels of serum T3 and T4, and is a more common disorder than clinical hypothyroidism. Patients with SCH have no symptoms (5). Correlation between SCH and increased risk of heart disease caused by atherosclerosis is shown in some studies, while it is not confirmed in some others. The relationship between lipid disorders, and SCH and thyroid hormones levels were also evaluated in patients in different studies, and different results were achieved (5,7).

 In the curent study, TCHOL levels in patients versus healthy cases did not differ except in the age group of 40 to 50 years that serum cholesterol levels were significantly higher in patients than the control group. In all patients, regardless of age group, the HDL cholesterol in females was significantly lower than that of the healthy females, but the difference between patients and healthy males was insignificant. The levels of LDL in patients were significantly higher than the healthy cases in the 2 genders. In the case of serum TG, there was no significant difference between the 2 groups. In overall, there was significant correlation between SCH, and decreased HDL level and increased LDL level regardless of age groups and gender, but there was no significant correlation between SCH, and increased levels of TG and TCHOL. These results might be associated with the increased risk of coronary artery disease in patients with SCH, because higher levels of LDL and lower levels of HDL might exacerbate the existing atherosclerosis. In addition, SCH can have an injurious effect on other cardiovascular risk factors such as development of hypercoagulable state (8), and impairment of ventricular function (9). Therefore, the concomitance of these factors could increase the risk of cardiovascular accidents in patients with SCH, and the thyroid replacement therapy could have beneficial effects on CVD risk in such patients. 

There are several studies in this area that show almost the same results of the current study. Data from a study by Hueston et al., (10) showed increased levels of TCHOL in patients with SCH versus the control group. But, after adjustment for confounding variables and the use of cholesterol-lowering drugs, no significant difference was observed between the 2 groups regarding lipid panel (10). In a study by Uzunlulu (11) et al. in Turkey, the prevalence of dyslipidemia in females with SCH was higher than that of the healthy ones (16.4% versus 8.5%). In 2011, Lai (12) found that the low levels of thyroid hormones in euthyroid patients were associated with dyslipidemia in the Chinese society. In the study by Luboshitzky (13) (2010), among lipid profile levels only TG was higher in patients with SCH, compared with healthy cases. The results of the study by Al Sayed et al., (14) (2006) on 34 patients with SCH and 20 healthy females demonstrated that TCHOL and LDL levels were significantly higher in patients than the control group; although the TG and HDL levels did not differ statistically between the 2 groups. In addition, there are several studies available with results contradictory to those of the current study (2, 15, 16). As an example, in the study by Azizi, (15) (2009)conducted on 1200 females from 4 different areas of Iran, 21.2% had SCH, out of which19% had dyslipidemia. In this study no significant correlation was observed in the rate of dyslipidemia between the females with SCH and healthy cases. 

## Conclusion

 As mentioned above, unlike clinical hypothyroidism known as secondary cause of hyperlipidemia (17, 18), contradictory results were achieved on the relationship between subclinical hypothyroidism and hyperlipidemia in different studies. The dissension between these studies may be explained by the following reasons: Different sample sizes in different studies, studies on different age groups, using different definitions of lipid profiles, different levels of blood lipid profiles according to race and ethnicity, and disregarding the diet (consumption of iodine salt and fat intake) and physical activity that could affect the lipid profiles. To solve these problems, it is recommended to conduct prospective studies with larger sample sizes, as cross sectional studies cannot show the causality between subclinical hypothyroidism and dyslipidemia. Also, it can be helpful to use homogeneous populations in terms of race, gender, age, and standard definitions for dyslipidemia for the community, and employ more sensitive tests to assess minor changes that may occur in cases with mild thyroid function disorders.

The effect of using levothyroxine to decrease the serum lipid levels in patients with SCH is not yet established. Two meta-analyses investigated the effects of thyroxine therapy on lipid profiles in patients with SCH (19, 20).

According to the current study and some other studies (5), biochemical screening for thyroid dysfunction is recommended for all patients with dyslipidemia; and underlying hypothyroidism should be treated in such patients. 

In addition, further studies with larger sample sizes are needed to find the effects of SCH on cardiovascular diseases, such as coronary artery disease and hypertension.
